# Genome-Wide Association Study Identifies Four Loci Associated with Eruption of Permanent Teeth

**DOI:** 10.1371/journal.pgen.1002275

**Published:** 2011-09-08

**Authors:** Frank Geller, Bjarke Feenstra, Hao Zhang, John R. Shaffer, Thomas Hansen, Ann-Louise Esserlind, Heather A. Boyd, Ellen A. Nohr, Nicholas J. Timpson, Ghazaleh Fatemifar, Lavinia Paternoster, David M. Evans, Robert J. Weyant, Steven M. Levy, Mark Lathrop, George Davey Smith, Jeffrey C. Murray, Jes Olesen, Thomas Werge, Mary L. Marazita, Thorkild I. A. Sørensen, Mads Melbye

**Affiliations:** 1 Department of Epidemiology Research, Statens Serum Institut, Copenhagen, Denmark; 2 Department of Human Genetics, Graduate School of Public Health, University of Pittsburgh, Pittsburgh, Pennsylvania, United States of America; 3 Research Institute of Biological Psychiatry, Mental Health Center Sct. Hans, Copenhagen University Hospital, Roskilde, Denmark; 4 Department of Neurology, Glostrup Hospital and the Danish Headache Center, Glostrup, Denmark; 5 Institute of Public Health, Aarhus University, Aarhus, Denmark; 6 Medical Research Council Centre for Causal Analyses in Translational Epidemiology, University of Bristol, Bristol, United Kingdom; 7 School of Social and Community Medicine, University of Bristol, Bristol, United Kingdom; 8 Department of Dental Public Health, School of Dental Medicine, University of Pittsburgh, Pittsburgh, Pennsylvania, United States of America; 9 Department of Preventive and Community Dentistry, University of Iowa, Iowa City, Iowa, United States of America; 10 Centre National de Génotypage, Evry, France; 11 Foundation Jean Dausset, Human Polymorphism Study Center (CEPH), Paris, France; 12 Department of Pediatrics, University of Iowa, Iowa City, Iowa, United States of America; 13 Center for Craniofacial and Dental Genetics, Department of Oral Biology, School of Dental Medicine, University of Pittsburgh, Pittsburgh, Pennsylvania, United States of America; 14 Institute of Preventive Medicine, Copenhagen University Hospital, Copenhagen, Denmark; Georgia Institute of Technology, United States of America

## Abstract

The sequence and timing of permanent tooth eruption is thought to be highly heritable and can have important implications for the risk of malocclusion, crowding, and periodontal disease. We conducted a genome-wide association study of number of permanent teeth erupted between age 6 and 14 years, analyzed as age-adjusted standard deviation score averaged over multiple time points, based on childhood records for 5,104 women from the Danish National Birth Cohort. Four loci showed association at *P*<5×10^−8^ and were replicated in four independent study groups from the United States and Denmark with a total of 3,762 individuals; all combined *P*-values were below 10^−11^. Two loci agreed with previous findings in primary tooth eruption and were also known to influence height and breast cancer, respectively. The two other loci pointed to genomic regions without any previous significant genome-wide association study results. The intronic SNP rs7924176 in *ADK* could be linked to gene expression in monocytes. The combined effect of the four genetic variants was most pronounced between age 10 and 12 years, where children with 6 to 8 delayed tooth eruption alleles had on average 3.5 (95% confidence interval: 2.9–4.1) fewer permanent teeth than children with 0 or 1 of these alleles.

## Author Summary

While genome-wide association studies (GWAS) initially focused on the disease under investigation, additional findings in secondary traits have shown further benefits of having extensive phenotype data at hand. Using records from the nationwide dental registry for children and genotype data from two GWAS, we were able to identify four genomic loci associated with permanent tooth eruption in children. Two of the identified genomic regions had no previous GWAS findings, whereas two loci were reported in the context of primary dentition. A follow-up in an on-going GWAS showed that rs7924176 also plays a substantial role in primary dentition. During the age period of permanent tooth eruption many important developmental processes take place. Thus, we suggest following up the four reported SNPs in other growth-related traits to further elucidate the genetic background of maturation.

## Introduction

Dental maturation is the process of exfoliation of primary teeth and eruption and calcification of permanent teeth that generally takes place between 6 and 13 years of age [1]. One commonly used measure of dental maturity is the number of permanent teeth erupted at a given age [2]. This is influenced by several factors, including gender [3]–[5], malnutrition [6], caries or trauma to the primary teeth [7], [8], ethnicity [9] and certain diseases [10]–[12]. In addition, many dental traits are known to be substantially influenced by genetic factors [13]–[15]. Although a recent study identified genetic variants for development of primary dentition in infancy [16], genetic factors influencing the eruption of permanent teeth have not been identified. To search for sequence variants associated with number of permanent teeth erupted, we carried out a GWAS in more than 5,100 women from the Danish National Birth Cohort (DNBC) [17], who had records in the nationwide dental registry for children (SCOR) and replicated the findings in more than 3,700 individuals from Denmark and the US (see Table S1 for description of study groups).

## Results

We analyzed the association between the number of permanent teeth erupted and 521,741 SNPs in 5,104 women with dental records from their childhood, and identified four loci strictly fulfilling genome-wide significance (*P*<5×10^−8^, see Figures S1 and S2 for quantile-quantile and Manhattan plots). No other region showed a *P*-value<5×10^−6^. To confirm the observed associations we genotyped and tested the most significant SNP at each of the four loci in additional Danish samples, as well as tested for *in silico* replication in a US study of dental caries. In the 3,762 additional individuals all SNPs replicated with *P*<10^−4^ and the combined *P*-values were <10^−11^ (Table 1).

**Table 1:** Initial, replication, and combined results for number of permanent teeth erupted between age 6 and 14 years, analyzed as age-adjusted standard deviation scores averaged over multiple time points.

SNP (effect/other allele)	rs12424086 C/T					rs4491709 T/C					rs2281845 T/C					rs7924176 G/A				
Study group	N	C freq.	Effect	SE	*P*-value	N	T freq.	Effect	SE	*P*-value	N	T freq.	Effect	SE	*P*-value	N	G freq.	Effect	SE	*P*-value
**Initial stage**																				
DNBC I GWAS	5099	0.177	−0.118	0.020	1.11E-08	5088	0.707	−0.109	0.017	6.51E-10	5097	0.381	−0.105	0.016	1.31E-10	5100	0.426	−0.094	0.016	6.58E-09
**Replication stage**																				
DNBC II	2162	0.166	−0.079	0.033	0.016	2171	0.717	−0.087	0.026	9.70E-04	2172	0.382	−0.099	0.025	5.43E-05	2172	0.420	−0.119	0.024	5.18E-07
DK Roskilde	671	0.186	−0.169	0.056	2.55E-03	690	0.698	−0.105	0.045	0.019	691	0.400	−0.165	0.042	1.09E-04	687	0.424	−0.089	0.043	0.040
USA	668	0.202	−0.130	0.067	0.051	669	0.732	−0.106	0.060	0.078	669	0.392	0.065	0.056	0.242	668	0.449	−0.160	0.051	1.80E-03
DK Glostrup	161	0.199	−0.020	0.094	0.833	161	0.730	−0.058	0.086	0.502	164	0.396	−0.175	0.069	0.012	163	0.439	−0.133	0.074	0.072
Replication combined	3662	0.177	−0.100	0.025	6.29E-05	3691	0.715	−0.091	0.021	9.09E-06	3696	0.388	−0.099	0.019	2.06E-07	3690	0.426	−0.120	0.019	1.25E-10
**All combined**	8761	0.177	−0.111	0.016	2.30E-12	8779	0.711	−0.102	0.013	2.16E-14	8793	0.384	−0.103	0.012	8.03E-17	8790	0.426	−0.105	0.012	5.64E-18
Heterogeneity *P*-value					0.528					0.947					0.015					0.678

Effect is given as the change in mean standard deviation score, so all effect alleles result in lower numbers of permanent teeth erupted. Alleles refer to the forward strand.

**Figure 1:**
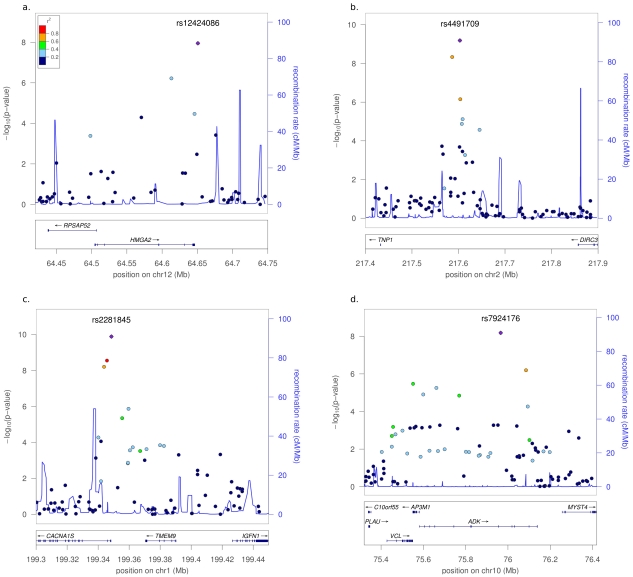
Plots of genetic regions associated with permanent tooth eruption. The figure shows a) chromosome 12q14.3, b) chromosome 2q35, c) chromosome 1q32.1, and d) chromosome 10q22.2 (generated with LocusZoom [38]). Displayed are the recombination rates over the region and *P*-values for the analyzed SNPs, the colors indicate the r^2^ with the most significant SNP.

Interestingly, two of the variants have been previously reported to affect primary tooth development [16]. First, rs12424086 on chromosome 12q14.3 had a suggestive *P*-value (5×10^−8^<*P*<5×10^−6^) for number of primary teeth at age 1 (*P* = 3.64×10^−6^) [16]. This SNP is about 120 kb downstream of *HMGA2* (see Figure 1 for genomic regions of the four associated SNPs) and is also in linkage disequilibrium (LD) with rs1042725 (r^2^ = 0.21 in HapMap Europeans, physical distance ∼6 kb), a SNP associated with adult and childhood height [18]. Second, rs4491709 on chromosome 2q35, is in LD with rs6435957 (r^2^ = 0.73 in the DNBC I study group, physical distance ∼17 kb), a SNP that showed suggestive evidence for an association with number of primary teeth at age 1 (*P* = 3.64×10^−7^) [16]. Furthermore, rs4491709 is in LD with rs13387042 (r^2^ = 0.34 in the DNBC I study group), which is associated with breast cancer [19]; the closest gene is *TNP1* (160 kb telomeric). For the genomic regions around the two other SNPs there were no previous significant GWAS results. The third SNP, rs2281845, on chromosome 1q32.1 is just upstream of *CACNA1S* (voltage-dependent calcium channel, L type), a gene subject to mutation screens for malignant hyperthermia [20], [21] and periodic paralysis [22], [23]. The LD block containing rs2281845 extends to *TMEM9* (transmembrane protein 9, physical distance 22 kb). The direction of the effect for rs2281845 in the US study group was - though not significant - opposite to the overall effect, which is most likely due to the relatively small sample size and greater variation in the phenotype (individual values are only based on one observation). The fourth SNP, rs7924176, on chromosome 10q22.2 is intronic in *ADK* (adenosine kinase), a gene that has been studied in the context of type 1 diabetes [24], and is located in a broader region showing linkage with Alzheimer's disease [25].

We also looked at all variants reportedly associated with tooth eruption in primary dentition (Table S2); among the 6 genome-wide significant SNPs, rs1956529 also showed a substantial effect on permanent tooth eruption, and three other loci had effects in the same direction with *P*-values between 0.01 and 0.17. The correlation between age at eruption of first tooth and permanent tooth eruption is evident (r = −0.408 in a subset of 1,442 children from the DNBC with questionnaire data on age at first tooth eruption and SCOR data on number of permanent teeth erupted; later ages at first tooth eruption going along with lower age-adjusted standard deviation scores (SDS) for number of permanent teeth erupted). We followed up all four SNPs associated with permanent tooth eruption in an on-going GWAS of primary dentition based on more than 6,000 individuals from the Avon Longitudinal Study of Parents and Children (ALSPAC) (Table S3 and Text S1). Rs7924176 also reached genome-wide significance in the analyses for number of primary teeth erupted at age 15 months and time to eruption of first tooth. The other three SNPs were at least nominally significant in the analysis of number of teeth erupted at age 15 months (*P* between 0.028 and 3.5×10^−6^), in all instances the same alleles associated with fewer teeth erupted in the respective dentition.

Given that both rs4491709 on chromosome 2q35 and rs1956529 on chromosome 14q24.1, which associated with tooth eruption in primary dentition [16], are close to breast cancer susceptibility loci, we checked all 19 SNPs associated with breast cancer in Caucasian study groups (*P*<5×10^−8^) [19], [26]–[32]. Only two of the other 18 loci were nominally significant (Table S4) without reaching a *P*<0.05/18. Thus, we could not find further evidence for a link to breast cancer.

Another trait of maturation, age at menarche, showed association with more than 30 SNPs in a recent GWA meta-analysis based on 87,802 women [33]. We checked the SNPs reported for age at menarche in our permanent tooth eruption GWAS (Table S5) and rs7821178 on chromosome 8q21.11 reached a *P*-value of 1×10^−4^, which is significant after adjustment for 42 SNPs tested. The allele increasing age at menarche also delayed tooth eruption, a pattern seen in all 6 SNPs with *P*<0.1 for permanent tooth eruption. This could be due to some genetic variants regulating maturation in a more general way, even though the correlation between age at menarche and permanent tooth eruption is modest (r = −0.095 in the combined DNBC I and DNBC II study groups, earlier age at menarche correlated with more permanent teeth erupted).

Results for the 180 SNPs recently reported to be associated with adult height [34] are given in Table S6. Apart from rs1351394, which is in LD with the identified SNP rs12424086 in the *HMGA2* region, rs6473015 (*P* = 6.1×10^−6^) was also significant after adjusting for the number of SNPs tested for height. This SNP is on chromosome 8q21.11 within 90 kb of rs7821178 (r^2^ = 0.78), the age at menarche variant with low *P*-value for permanent tooth eruption. The correlation between permanent tooth eruption and adult height is modest (r = 0.074 in the combined DNBC I and DNBC II study groups, more permanent teeth erupted correlated with increased adult height) and there is no consistent direction among the height SNPs reaching nominal significance for permanent tooth eruption.

The correlation between age at menarche and adult height (r = 0.099, earlier age at menarche correlated with decreased adult height) is well known from the literature [35], but is opposite to what would be expected just based on the correlation results for permanent tooth eruption. Even though all correlations are modest, they underline that the interplay between these three growth and maturation traits is not straightforward.

To follow-up on the (potential) links to age at menarche, height and breast cancer, we contacted the latest published GWAS for these traits [26], [33], [34] and results for the reported four SNPs are displayed in Tables S7, S8, S9.

For age at menarche there is no indication that the four SNPs play a role (lowest *P* = 0.15) based on results from the meta-analysis with 87,802 women of European descent (Table S7).

For height, results based on 133,000 individuals showed the expected signal for rs12424086 in the *HMGA2* region, and rs4491709 had a modest effect that reached nominal significance (*P* = 0.02, Table S8).

Currently, several GWAS on breast cancer have been conducted, with the latest having a combined 2,839 cases and 3,507 controls at the initial GWAS stage [26]. This group provided us with results for 1,693 cases vs. 5,588 controls (Table S9) and rs4491709 (which is in LD with the known breast cancer risk SNP rs6435957 (r^2^ = 0.73), reached nominal significance (*P* = 0.024), for the three other regions the lowest *P* was 0.096.

We investigated association with expression levels of nearby genes in an expression database for monocytes [36]. The four SNPs were not genotyped directly, but several SNPs in LD with rs2281845 showed significant association with expression of *TMEM9*, and the SNP with the strongest LD (rs6667912, r^2^ = 0.46 in HapMap Europeans) had a *P*-value of 6×10^−29^. Similarly, we observed association of rs7924176 with expression of *ADK* via rs1874152 (r^2^ = 0.71 in HapMap Europeans) at a *P*-value of 2×10^−42^.

**Figure 2:**
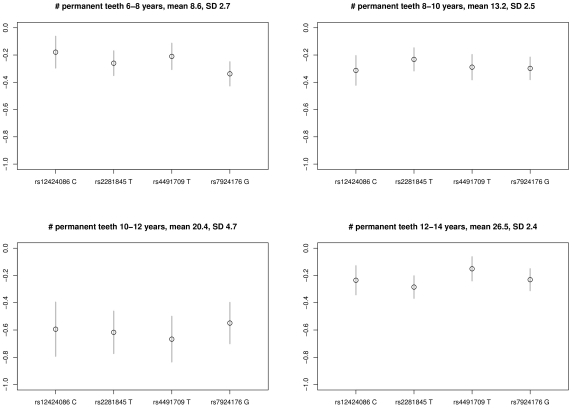
Per allele effect of the four variants on number of permanent teeth erupted based on data for women from the DNBC I & II study groups at age 6–8 years (N = 5,865), 8–10 years (N = 6,548), 10–12 years (N = 6,919), and 12–14 years (N = 7,059).

**Figure 3:**
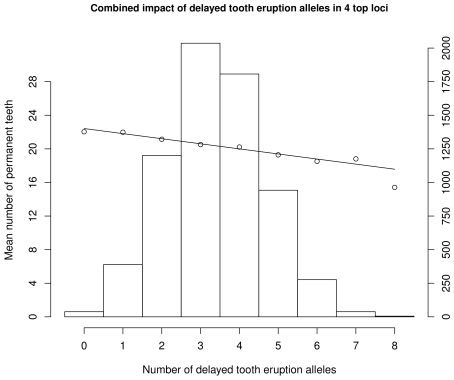
Combined distribution of number of delayed permanent tooth eruption alleles and combined effects for all four identified SNPs at age 10–12 years (N = 6,919).

The women from the DNBC provided a relatively homogeneous group with similar mean number of individual observations. Therefore, we split the time period from age 6 to 14 years into four two-year periods to see the effect of the variants at different ages (Figure 2). All variants showed significant effects on the number of teeth erupted in all four age periods, including the age group 12 to 14 years in which the permanent dentition was nearly or fully erupted (mean number of erupted teeth is 26.5). The highest variation was observed in the age group 10 to 12 years (standard deviation (SD) 4.7 teeth), and went along with the strongest per allele effect estimates (−0.55 to −0.67 teeth). Considering the combined number of delayed tooth eruption alleles, the 4.8% of children with 6 to 8 delayed eruption alleles had on average 18.5 (SD 4.5) permanent teeth compared to 22.0 (SD 4.2) in the 6.3% of children with 0 or 1 risk allele (Figure 3). Effect estimates in the three other age categories ranged from −0.15 to −0.34 teeth per allele. The variance explained by the four variants combined ranged from 1.5% (age 12 to 14) to 3.0% (age 10 to 12).

Permanent tooth eruption happens earlier in girls than in boys, so we checked for gender differences in the replication groups, but there was no indication of heterogeneous effects between sexes (results not shown). However, the low number of male individuals in the study means that we only have limited power to detect effect differences between sexes.

## Discussion

We carried out the first GWAS for normal variation in the timing of permanent tooth eruption. Using longitudinal dental data between age 6 and 14 years, we identified four loci with robust associations. The main strengths of our study are: i) the comprehensive data set on dental exams during childhood, which allowed us to generate a mean SDS for the number of permanent teeth erupted, ii) the Danish replication groups with comprehensive phenotype data from the same database as the initial study, and iii) the substantial sample size of more than 3,700 individuals at the replication stage. The main limitations of the study are i) the lack of corresponding data on primary dentition, which is owed to the fact that there is usually no need to present children to the dentist in the time period where primary teeth erupt, ii) the lack of other growth and maturation traits, because such data are not collected in connection with visits to the dentist and are not readily available otherwise.

The connection of our study to primary tooth eruption is obvious with rs7924176 reaching genome-wide significance in the analysis of number of permanent teeth erupted at age 15 months in the ALSPAC data, and the three other loci being at least nominally significant. Looking at the 6 SNPs previously reported as genome-wide significant (*P*<5×10^−8^) for primary dentition, rs1956529 in the *RAD51L1* region showed association with permanent dentition (p = 3.6×10^−5^) and three SNPs had *P*-values between 0.01 and 0.17, with the effect going in the same direction. Thus, maybe all these SNPs are relevant in both tooth eruption periods, just with substantial variation in the strength of association between periods. Children with data for both tooth eruption periods are necessary to see whether SNPs with strong effects on primary tooth eruption have an independent effect in permanent dentition. On the other hand, there was no association with permanent tooth eruption for the two correlated SNPs in the *EDA* region (both *P*>0.4 and effect in opposite direction), which means that there are also substantial differences between the genetic mechanisms driving primary and permanent tooth eruption.

The two identified SNPs previously reported in primary dentition are in LD with genome-wide significant SNPs in adult and childhood height (chromosome 12q14.3) and breast cancer (chromosome 2q35). Additional studies will be necessary to determine whether similar or different mechanisms explain the associations in these two regions.

We analyzed expression data for monocytes to investigate functional implications of the four SNPs. Rs2281845 and rs7924176 could be linked to expression of *TMEM9* and *ADK*, respectively, with rs7924176 being intronic in *ADK*. However, the function of *TMEM9* is unknown whereas *ADK* regulates the concentrations of extracellular adenosine and intracellular adenine nucleotides, providing no further insight into the potential role of these genes in tooth development.

Dental maturation was found to proceed rather independently of other forms of biological maturation in epidemiologic studies [1], [37] and our comprehensive comparisons with other GWAS findings for adult height and age at menarche showed only modest overlap between associated variants. Investigating permanent tooth eruption in 180 height loci and 42 age at menarche loci revealed three SNPs that were significant after adjustment for the number of variants tested for each trait, with the first signal being the height SNP in the previously discussed chromosome 12q14.3 region. The two other SNPs are correlated and located on chromosome 8q close to *PXMP3* (peroxin 2), with GWAS findings for both age at menarche and adult height, suggesting that this region is of general importance in growth and maturation.

Age at menarche is currently the only pubertal trait with GWAS findings, leaving the genetic background of pubertal growth and maturation poorly understood. A focused investigation of the four SNPs in other puberty-related traits, e.g. skeletal maturation, Tanner score and growth spurt, could reveal whether these variants regulate maturation in a global way or are rather specific to dental maturation.

## Methods

### Study Groups

Basic characteristics of the study populations are provided as Table S1. For all study groups, all individuals with available information on permanent tooth eruption were analyzed, regardless of the phenotype they were recruited for.

#### DNBC I and II

The initial stage of the study (DNBC I, N = 5,104) was conducted in the context of the Danish National Birth Cohort, a project including mothers and their children from 101,042 pregnancies recruited in the years 1996–2002 [17]. Blood samples from mothers were obtained two times during pregnancy (around gestational weeks 8 and 24) and stored in a biobank at Statens Serum Institut as buffy coats. The genotype data were derived from two on-going GWAS of preterm birth [39] and obesity [40]. Additional 2,229 mothers (DNBC II) in the replication stage were recruited for studies of preeclampsia and psoriasis.

The study protocol was approved by the Danish Scientific Ethical Committee and the Danish Data Protection Agency for all subjects.

#### Denmark—Roskilde

The Danish study group from Roskilde (N = 695) included individuals drawn from the Danish Psychiatric Biobank, a national research platform that recruits individuals for gene-environment association studies [41]. DNA was obtained from whole blood. All participants have given written informed consent and the study protocol was approved by the Danish Scientific Ethical Committee and the Danish Data Protection Agency.

#### United States

The US replication sample (N = 669) included individuals recruited from the Center for Oral Health Research in Appalachia [42], [43] and the Iowa Fluoride Study [44]–[47], two initiatives designed to study oral health outcomes and included as part of the Gene Environment Association Studies consortium (GENEVA). DNA was obtained from blood, buccal swab, mouthwash, and saliva samples. All participants provided verbal assent with parental written consent, and all study protocols were approved by the pertinent Institutional Review Boards at the University of Pittsburgh, West Virginia University, and University of Iowa.

#### Denmark—Glostrup

The Danish study group from Glostrup (N = 169) included individuals recruited for a study of migraine and were selected from the Danish National Patient Register and from case files from neurological clinics [48]. All participants have given written informed consent and the study protocol was approved by the Danish Scientific Ethical Committee and the Danish Data Protection Agency.

### Genotyping and Quality Control

Genotyping for the DNBC I and the US group was performed with Illumina (Illumina, SanDiego, CA, USA) Human 660W-Quad (preterm birth) and 610-Quad (obesity and US) bead chips. Single SNP genotyping of rs2281845, rs4491709, rs7924176 and rs12424086 for the Danish replication samples was carried out at deCODE genetics using the Centaurus (Nanogen, Bothell, WA, USA) platform.

The initial GWAS was based on SNPs that passed quality control on both chips, SNPs were excluded based on a missing rate >2%, deviation from Hardy-Weinberg equilibrium (*P*<10^−4^) or minor allele frequency <0.5%, individuals with more than 5% missing genotypes were also excluded. The genotypes for the four selected SNPs in the replication stage did not show deviation from Hardy-Weinberg equilibrium or missing rates >2%.

### Phenotype

Dental data for all Danish individuals were retrieved from the nationwide dental registry for children, SCOR, which was established in 1972 alongside the initiation of free municipal dental services to Danish children and adolescents from birth to the age of 18 years [49]. Data were reported annually for all children until January 1^st^ 1993, from which date reporting was only mandatory for 5-, 7-, 12-, and 15-year-old children [50].

All participants for the US sample underwent intraoral examinations by trained dental clinicians to collect phenotype data including number of erupted permanent teeth.

### Statistical Analysis

The study combined all observations between age 6 and 14 years (starting with the 6^th^ and stopping with the 14^th^ birthday), the time period when eruption of permanent dentition usually occurs. For each visit to the dentist the total number of permanent teeth (excluding third molars) was recorded, and regressed on age. The resulting residuals were then standardized, and for each individual the mean residual across all available records was used as phenotype. This approach was motivated by a recent study, which demonstrated that averaging over multiple records increases the power in quantitative trait analysis [51]. For the DNBC I study group, we limited analyses to SNPs passing quality control on both chips and analyzed the two subgroups (preterm birth study/obesity study) together. Initial GWAS analysis of mean standardized residuals was carried out by applying the Wald test under an additive genetic model (also for chromosome X since all analyzed individuals are female) as implemented in PLINK [52]. We selected only SNPs with *P*<5×10^−8^ for replication, because SNPs in other regions did not show *P*-values<5×10^−6^. With the initial GWAS reaching genome-wide significance, the replication was basically of technical nature, aiming to confirm the results for the four SNPs with a second genotyping method. The criterion for overall significance remained *P*<5×10^−8^, but the additional study groups should not show great heterogeneity in terms of effect estimates and therefore an improvement of the combined *P*-values was expected.

*P*-values from the studies based on chip-typed individuals were corrected by applying genomic control (estimated genomic inflation factors for the DNBC I and US study groups were 1.05 and 1.01, respectively). Combined effects and *P*-values were calculated using the inverse variance method as implemented in METAL [53]. Though the number of permanent teeth was not normally distributed and the distribution of mean standardized residuals remained somewhat skewed, parametric tests were nevertheless carried out to determine the effect of the variants. However, to protect against false positive findings due to the phenotype distribution, we additionally carried out a non-parametric analysis of our top SNPs from the four significant loci using Kruskal-Wallis tests in R and calculated combined *P*-values based on weighted Z-scores (Table S10). Despite the reduced power of the non-parametric analysis (which also does not account for the trend observed over the genotype groups), all SNPs reached genome-wide significance (*P*<5×10^−8^).

### Population Structure and Relatedness

To control for possible population substructure, we performed multidimensional scaling analysis (as implemented in PLINK [52]) on the discovery data, using independent autosomal SNPs with missing call rates <1%, minor allele frequency >5%, and Hardy-Weinberg *P*-value>0.05. We utilized PLINK's LD pruning function to remove short and long-range LD. The resulting 23,111 SNPs were analyzed along with founder genotypes from 11 HapMap phase III reference populations. As expected most discovery samples clustered near the Caucasian populations from Utah and Tuscany, but 61 individuals fell more than 4 standard deviations away from the discovery set mean on one or more of the first five dimensions and were excluded from further analysis.

For all Danish replication sets, we obtained birthplace information from the Danish Civil Registry [54], and only included individuals who were born in Scandinavia and whose parents were not born outside of Europe. Data from the Danish Family Relations Database were used to exclude all individuals who were first or second degree relatives to an individual in the initial set or another subject already in the replication set.

The samples from the Denmark Roskilde and Glostrup groups were additionally genotyped on an ancestry informative microsatellite panel to confirm self-reported Danish European ethnicity, and individuals with <90% European ancestry were removed.

In the US study group we performed multidimensional scaling analysis to account for possible population substructure, resulting in the exclusion of 45 individuals. Recruitment was partly family-based (mainly sib ships), and we decided to keep one individual per pedigree based on the examination being closest to 10.0 years (the midpoint of the investigated age interval), leading to the exclusion of 132 individuals.

### Imputation for the DNBC I Study Group

In order to allow for comparisons with previous GWAS on related phenotypes, we separately imputed genotypes for the two GWAS included in the DNBC I study group applying MACH [55]. The imputed genotypes were analyzed separately and meta-analyzed with METAL [53].

### Web Resources

ALSPAC http://www.bristol.ac.uk/alspac/;

British 1958 Birth Cohort: http://www.b58cgene.sgul.ac.uk/;

DNBC: http://dnbc.dk/;

eQTL database: http://eqtl.uchicago.edu/cgi-bin/gbrowse/eqtl/;

GENEVA: http://www.genevastudy.org;

GIANT meta-analysis: http://www.broadinstitute.org/collaboration/giant/index.php/Main_Page; HapMap: http://hapmap.ncbi.nlm.nih.gov/;

R: http://www.r-project.org/;

## Supporting Information

Figure S1 Quantile-quantile plot for GWAS of permanent tooth eruption between age 6 and 14 years (analyzed as age-adjusted standard deviation scores averaged over multiple time points) in 5,104 women from the DNBC I study group.
(TIF)

Figure S2 Manhattan plot for GWAS of permanent tooth eruption between age 6 and 14 years (analyzed as age-adjusted standard deviation scores averaged over multiple time points) in 5,104 women from the DNBC I study group.
(TIF)

Table S1 Descriptive statistics of study groups. a) Basic description of study groups, and b) detailed distribution of number of exams by study group.
(DOC)

Table S2 Results for GWAS of permanent tooth eruption between age 6 and 14 years in 5,104 women from the DNBC for all variants previously reported for primary dentition [16].
(DOC)

Table S3 Primary tooth eruption analysis for the identified SNPs in the ALSPAC data. Results are presented for a) number of primary teeth erupted at age 15 months based on 6,609 individuals and b) time to eruption of first tooth based on 5,998 individuals.
(DOC)

Table S4 Results from GWAS of permanent tooth eruption between age 6 and 14 years in 5,104 women from the DNBC for 19 variants previously reported with *P*<5×10^−8^ for breast cancer in Caucasians [19], [26]–[32].
(DOC)

Table S5 Results for GWAS of permanent tooth eruption between age 6 and 14 years in 5,104 women from the DNBC for all 42 variants previously reported for age at menarche [33].
(DOC)

Table S6 Results from GWAS of permanent tooth eruption between age 6 and 14 years in 5,104 women from the DNBC for 180 variants previously reported for adult height [34].
(DOC)

Table S7 Age at menarche results for the four identified SNPs based on the GWAS meta-analysis with 87,802 women.
(DOC)

Table S8 Height results for the four identified SNPs based on the GWAS meta-analysis with 183,727 individuals.
(DOC)

Table S9 Breast cancer results for the four identified SNPs based on the recent British Breast Cancer GWAS.
(DOC)

Table S10 Kruskal-Wallis tests for data in Table 1.
(DOC)

Text S1 ALSPAC GWAS for primary dentition.
(DOC)

## References

[1] Beunen GP, Rogol AD, Malina RM (2006). Indicators of biological maturation and secular changes in biological maturation.. Food Nutr Bull.

[2] Hagg U, Taranger J (1985). Dental development, dental age and tooth counts.. Angle Orthod.

[3] Helm S, Seidler B (1974). Timing of permanent tooth emergence in Danish children.. Community Dent Oral Epidemiol.

[4] Leroy R, Bogaerts K, Lesaffre E, Declerck D (2003). The emergence of permanent teeth in Flemish children.. Community Dent Oral Epidemiol.

[5] Eskeli R, Laine-Alava MT, Hausen H, Pahkala R (1999). Standards for permanent tooth emergence in Finnish children.. Angle Orthod.

[6] Psoter W, Gebrian B, Prophete S, Reid B, Katz R (2008). Effect of early childhood malnutrition on tooth eruption in Haitian adolescents.. Community Dent Oral Epidemiol.

[7] Leroy R, Bogaerts K, Lesaffre E, Declerck D (2003). Impact of caries experience in the deciduous molars on the emergence of the successors.. Eur J Oral Sci.

[8] Korf SR (1965). The eruption of permanent central incisors following premature loss of their antecedents.. ASDC J Dent Child.

[9] Chaillet N, Nystrom M, Demirjian A (2005). Comparison of dental maturity in children of different ethnic origins: international maturity curves for clinicians.. J Forensic Sci.

[10] Lal S, Cheng B, Kaplan S, Softness B, Greenberg E (2008). Accelerated tooth eruption in children with diabetes mellitus.. Pediatrics.

[11] Lehtinen A, Oksa T, Helenius H, Ronning O (2000). Advanced dental maturity in children with juvenile rheumatoid arthritis.. Eur J Oral Sci.

[12] Suri L, Gagari E, Vastardis H (2004). Delayed tooth eruption: pathogenesis, diagnosis, and treatment. A literature review.. Am J Orthod Dentofacial Orthop.

[13] Townsend G, Hughes T, Luciano M, Bockmann M, Brook A (2009). Genetic and environmental influences on human dental variation: a critical evaluation of studies involving twins.. Arch Oral Biol.

[14] Pelsmaekers B, Loos R, Carels C, Derom C, Vlietinck R (1997). The genetic contribution to dental maturation.. J Dent Res.

[15] Hughes TE, Bockmann MR, Seow K, Gotjamanos T, Gully N (2007). Strong genetic control of emergence of human primary incisors.. J Dent Res.

[16] Pillas D, Hoggart CJ, Evans DM, O'Reilly PF, Sipila K (2010). Genome-wide association study reveals multiple loci associated with primary tooth development during infancy.. PLoS Genet.

[17] Olsen J, Melbye M, Olsen SF, Sorensen TI, Aaby P (2001). The Danish National Birth Cohort–its background, structure and aim.. Scand J Public Health.

[18] Weedon MN, Lettre G, Freathy RM, Lindgren CM, Voight BF (2007). A common variant of HMGA2 is associated with adult and childhood height in the general population.. Nat Genet.

[19] Stacey SN, Manolescu A, Sulem P, Rafnar T, Gudmundsson J (2007). Common variants on chromosomes 2q35 and 16q12 confer susceptibility to estrogen receptor-positive breast cancer.. Nat Genet.

[20] Carpenter D, Ringrose C, Leo V, Morris A, Robinson RL (2009). The role of CACNA1S in predisposition to malignant hyperthermia.. BMC Med Genet.

[21] Levano S, Vukcevic M, Singer M, Matter A, Treves S (2009). Increasing the number of diagnostic mutations in malignant hyperthermia.. Hum Mutat.

[22] Lin SH, Hsu YD, Cheng NL, Kao MC (2005). Skeletal muscle dihydropyridine-sensitive calcium channel (CACNA1S) gene mutations in chinese patients with hypokalemic periodic paralysis.. Am J Med Sci.

[23] Ng WY, Lui KF, Thai AC, Cheah JS (2004). Absence of ion channels CACN1AS and SCN4A mutations in thyrotoxic hypokalemic periodic paralysis.. Thyroid.

[24] Lucarelli P, Scacchi R, Corbo RM, Palmarino R, Orsini M (1978). Genetic polymorphisms in juvenile-onset diabetes.. Hum Hered.

[25] Grupe A, Li Y, Rowland C, Nowotny P, Hinrichs AL (2006). A scan of chromosome 10 identifies a novel locus showing strong association with late-onset Alzheimer disease.. Am J Hum Genet.

[26] Fletcher O, Johnson N, Orr N, Hosking FJ, Gibson LJ (2011). Novel breast cancer susceptibility locus at 9q31.2: results of a genome-wide association study.. J Natl Cancer Inst.

[27] Li J, Humphreys K, Heikkinen T, Aittomaki K, Blomqvist C (2011). A combined analysis of genome-wide association studies in breast cancer.. Breast Cancer Res Treat.

[28] Antoniou AC, Wang X, Fredericksen ZS, McGuffog L, Tarrell R (2010). A locus on 19p13 modifies risk of breast cancer in BRCA1 mutation carriers and is associated with hormone receptor-negative breast cancer in the general population.. Nat Genet.

[29] Turnbull C, Ahmed S, Morrison J, Pernet D, Renwick A (2010). Genome-wide association study identifies five new breast cancer susceptibility loci.. Nat Genet.

[30] Thomas G, Jacobs KB, Kraft P, Yeager M, Wacholder S (2009). A multistage genome-wide association study in breast cancer identifies two new risk alleles at 1p11.2 and 14q24.1 (RAD51L1).. Nat Genet.

[31] Hunter DJ, Kraft P, Jacobs KB, Cox DG, Yeager M (2007). A genome-wide association study identifies alleles in FGFR2 associated with risk of sporadic postmenopausal breast cancer.. Nat Genet.

[32] Easton DF, Pooley KA, Dunning AM, Pharoah PD, Thompson D (2007). Genome-wide association study identifies novel breast cancer susceptibility loci.. Nature.

[33] Elks CE, Perry JR, Sulem P, Chasman DI, Franceschini N (2010). Thirty new loci for age at menarche identified by a meta-analysis of genome-wide association studies.. Nat Genet.

[34] Lango Allen H, Estrada K, Lettre G, Berndt SI, Weedon MN (2010). Hundreds of variants clustered in genomic loci and biological pathways affect human height.. Nature.

[35] Onland-Moret NC, Peeters PH, van Gils CH, Clavel-Chapelon F, Key T (2005). Age at menarche in relation to adult height: the EPIC study.. Am J Epidemiol.

[36] Zeller T, Wild P, Szymczak S, Rotival M, Schillert A (2010). Genetics and beyond–the transcriptome of human monocytes and disease susceptibility.. PLoS One.

[37] Demirjian A, Buschang PH, Tanguay R, Patterson DK (1985). Interrelationships among measures of somatic, skeletal, dental, and sexual maturity.. Am J Orthod.

[38] Pruim RJ, Welch RP, Sanna S, Teslovich TM, Chines PS (2010). LocusZoom: regional visualization of genome-wide association scan results.. Bioinformatics.

[39] Cornelis MC, Agrawal A, Cole JW, Hansel NN, Barnes KC (2010). The Gene, Environment Association Studies consortium (GENEVA): maximizing the knowledge obtained from GWAS by collaboration across studies of multiple conditions.. Genet Epidemiol.

[40] Nohr EA, Timpson NJ, Andersen CS, Davey SG, Olsen J (2009). Severe obesity in young women and reproductive health: the Danish National Birth Cohort.. PLoS One.

[41] Hansen T, Olsen L, Lindow M, Jakobsen KD, Ullum H (2007). Brain expressed microRNAs implicated in schizophrenia etiology.. PLoS One.

[42] Wang X, Shaffer JR, Weyant RJ, Cuenco KT, DeSensi RS (2010). Genes and their effects on dental caries may differ between primary and permanent dentitions.. Caries Res.

[43] Polk DE, Weyant RJ, Crout RJ, McNeil DW, Tarter RE (2008). Study protocol of the Center for Oral Health Research in Appalachia (COHRA) etiology study.. BMC Oral Health.

[44] Franzman MR, Levy SM, Warren JJ, Broffitt B (2004). Tooth-brushing and dentifrice use among children ages 6 to 60 months.. Pediatr Dent.

[45] Marshall TA, Levy SM, Broffitt B, Warren JJ, Eichenberger-Gilmore JM (2003). Dental caries and beverage consumption in young children.. Pediatrics.

[46] Levy SM, Warren JJ, Broffitt B (2003). Patterns of fluoride intake from 36 to 72 months of age.. J Public Health Dent.

[47] Warren JJ, Levy SM, Kanellis MJ (2002). Dental caries in the primary dentition: assessing prevalence of cavitated and noncavitated lesions.. J Public Health Dent.

[48] Anttila V, Stefansson H, Kallela M, Todt U, Terwindt GM (2010). Genome-wide association study of migraine implicates a common susceptibility variant on 8q22.1.. Nat Genet.

[49] Helm S (1973). Recording system for the Danish Child Dental Health Services.. Community Dent Oral Epidemiol.

[50] Hansen I, Foldspang A, Poulsen S (2001). Use of a national database for strategic management of municipal oral health services for Danish children and adolescents.. Community Dent Oral Epidemiol.

[51] Rasmussen-Torvik LJ, Alonso A, Li M, Kao W, Kottgen A (2010). Impact of repeated measures and sample selection on genome-wide association studies of fasting glucose.. Genet Epidemiol.

[52] Purcell S, Neale B, Todd-Brown K, Thomas L, Ferreira MA (2007). PLINK: a tool set for whole-genome association and population-based linkage analyses.. Am J Hum Genet.

[53] Willer CJ, Li Y, Abecasis GR (2010). METAL: fast and efficient meta-analysis of genomewide association scans.. Bioinformatics.

[54] Pedersen CB, Gotzsche H, Moller JO, Mortensen PB (2006). The Danish Civil Registration System. A cohort of eight million persons.. Dan Med Bull.

[55] Li Y, Willer CJ, Ding J, Scheet P, Abecasis GR (2006). MaCH: using sequence and genotype data to estimate haplotypes and genotype data to estimate haplotypes and unobserved genotypes.. Genet Epidemiol.

