# Supplementary figures and images for: Genome-Wide Association Study Identifies Four Loci Associated with Eruption of Permanent Teeth
Source: PLoS Genet. 2011 Sep 8;7(9):e1002275. doi: 10.1371/journal.pgen.1002275 (PMC3169538; doi:10.1371/journal.pgen.1002275)

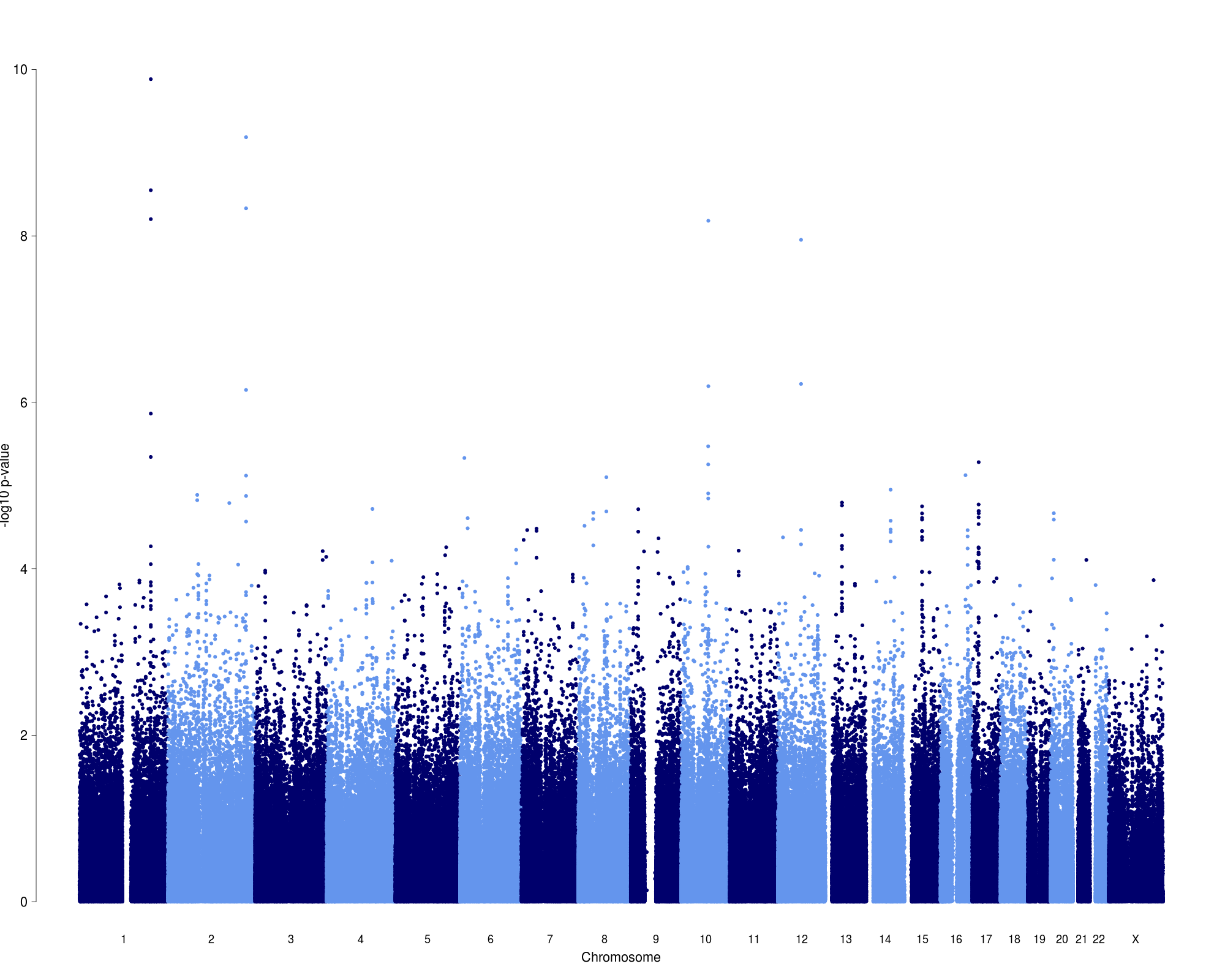

Supplement: Figure S2 — Manhattan plot for GWAS of permanent tooth eruption between age 6 and 14 years (analyzed as age-adjusted standard deviation scores averaged over multiple time points) in 5,104 women from the DNBC I study group. (TIF) [file pgen.1002275.s002.tif]
