# Supplementary material for: Genome-Wide Association Study Identifies Four Loci Associated with Eruption of Permanent Teeth
Source: PLoS Genet. 2011 Sep 8;7(9):e1002275. doi: 10.1371/journal.pgen.1002275 (PMC3169538; doi:10.1371/journal.pgen.1002275)
Supplement: Table S1 — Descriptive statistics of study groups. a) Basic description of study groups, and b) detailed distribution of number of exams by study group. (DOC) [file pgen.1002275.s003.doc]

**Table S1**: Descriptive statistics of study groups. a) Basic description of study groups, and b) detailed distribution of number of exams by study group.

| **1a** |  |  |  |  |
| --- | --- | --- | --- | --- |
| **Study group** | **N** | **% females** | **Mean number of exams (SD)** | **Mean year of birth (SD)** |
| **Initial stage** |  |  |  |  |
| DNBC I GWAS | 5104 | 100.0% | 6.39 (1.64) | 1971 (3.7) |
| **Replication stage** |  |  |  |  |
| DNBC II | 2229 | 100.0% | 6.33 (1.73) | 1971 (3.6) |
| DK Roskilde | 695 | 41.2% | 5.96 (1.90) | 1976 (7.3) |
| USA | 669 | 51.3% | 1 (-----) | 1994 (1.9) |
| DK Glostrup | 169 | 71.0% | 5.85 (1.99) | 1972 (6.9) |

| **1b** |  |  |  |  |  |
| --- | --- | --- | --- | --- | --- |
| **Number of exams** | **DNBC I** | **DNBC II** | **DK Roskilde** | **USA** | **DK Glostrup** |
| 1 | 83 | 45 | 26 | 669 | 7 |
| 2 | 124 | 69 | 30 |  | 8 |
| 3 | 169 | 87 | 32 |  | 11 |
| 4 | 253 | 108 | 54 |  | 15 |
| 5 | 467 | 184 | 79 |  | 13 |
| 6 | 987 | 436 | 136 |  | 34 |
| 7 | 1755 | 768 | 203 |  | 47 |
| 8 | 1156 | 477 | 123 |  | 32 |
| 9 | 103 | 51 | 12 |  | 2 |
| 10 | 7 | 1 |  |  |  |
| 11 |  | 1 |  |  |  |
| 12 |  | 0 |  |  |  |
| 13 |  | 1 |  |  |  |
| 14 |  | 1 |  |  |  |
