# Supplementary material for: Genome-Wide Association Study Identifies Four Loci Associated with Eruption of Permanent Teeth
Source: PLoS Genet. 2011 Sep 8;7(9):e1002275. doi: 10.1371/journal.pgen.1002275 (PMC3169538; doi:10.1371/journal.pgen.1002275)
Supplement: Table S2 — Results for GWAS of permanent tooth eruption between age 6 and 14 years in 5,104 women from the DNBC for all variants previously reported for primary dentition [16]. (DOC) [file pgen.1002275.s004.doc]

**Table S2**: Results for GWAS of permanent tooth eruption between age 6 and 14 years in 5,104 women from the DNBC for all variants previously reported for primary dentition [16].

| **SNP** | **Chromosome** | **Bp** | **Effect allele** | **Other allele** | **Effect allele freq** | **N** | **Effect (SDS)** | **SE** | ***P*-value** | **Direction** |
| --- | --- | --- | --- | --- | --- | --- | --- | --- | --- | --- |
| rs8079702 | 17 | 65702421 | G | A | 0.432 | 5102 | -0.028 | 0.015 | 0.072 | + |
| rs4844096 | X | 68722043 | A | G | 0.424 | 5101 | -0.012 | 0.016 | 0.448 | - |
| rs5936487 | X | 68809641 | G | A | 0.426 | 5102 | 0.006 | 0.016 | 0.691 | - |
| rs10506525 | 12 | 64069645 | A | G | 0.360 | 5102 | 0.042 | 0.016 | 0.012 | + |
| rs9674544 | 17 | 44439710 | G | A | 0.475 | 5104 | -0.022 | 0.015 | 0.163 | + |
| rs1956529 | 14 | 67858677 | G | A | 0.367 | 5097 | 0.067 | 0.016 | 3.61E-05 | + |
| rs6435957 | 2 | 217586454 | C | T | 0.325 | 5104 | 0.099 | 0.017 | 4.66E-09 | + |
| rs9386463 | 6 | 106200750 | A | G | 0.476 | 5102 | 0.005 | 0.015 | 0.752 | + |
| rs6504340 | 17 | 43972018 | A | G | 0.207 | 5101 | 0.005 | 0.019 | 0.806 | + |
| rs2817937 | 6 | 121140156 | G | A | 0.094 | 5103 | 0.016 | 0.026 | 0.548 | - |
| rs12424086 | 12 | 64650776 | C | T | 0.177 | 5099 | -0.118 | 0.020 | 1.11E-08 | + |

Effects and frequency are given for the minor allele in the DNBC I group, the direction column indicates whether the allele associated with lower number of primary teeth (longer time to first tooth eruption) has a (consistent) negative effect in number of permanent teeth erupted (+) or not (-). SNPs above the line reached genome-wide significance in primary dentition, SNPs below the line were suggestive. Alleles refer to the forward strand.
