# Supplementary material for: Genome-Wide Association Study Identifies Four Loci Associated with Eruption of Permanent Teeth
Source: PLoS Genet. 2011 Sep 8;7(9):e1002275. doi: 10.1371/journal.pgen.1002275 (PMC3169538; doi:10.1371/journal.pgen.1002275)
Supplement: Table S3 — Primary tooth eruption analysis for the identified SNPs in the ALSPAC data. Results are presented for a) number of primary teeth erupted at age 15 months based on 6,609 individuals and b) time to eruption of first tooth based on 5,998 individuals. (DOC) [file pgen.1002275.s005.doc]

**Table S3**: Primary tooth eruption analysis for the identified SNPs in the ALSPAC data. Results are presented for a) number of primary teeth erupted at age 15 months based on 6,609 individuals and b) time to eruption of first tooth based on 5,998 individuals.

| **3a** | | | | | | | |
| --- | --- | --- | --- | --- | --- | --- | --- |
| **SNP** | **Effect allele** | **Other allele** | **Effect allele freq** | **R-squared** | **Effect** | **SE** | ***P*-value** |
| rs7924176 | G | A | 0.421 | 0.997 | -0.248 | 0.031 | 8.77E-16 |
| rs12424086 | C | T | 0.201 | 0.999 | -0.177 | 0.038 | 3.46E-06 |
| rs4491709 | T | C | 0.715 | 0.999 | -0.088 | 0.034 | 8.96E-03 |
| rs2281845 | T | C | 0.397 | 0.956 | -0.071 | 0.032 | 0.028 |
|  | | | | | | | |
| **3b** |  |  |  |  |  |  |  |
| **SNP** | **Effect allele** | **Other allele** | **Effect allele freq** | **R-squared** | **Effect** | **SE** | ***P*-value** |
| rs7924176 | G | A | 0.421 | 0.997 | 0.261 | 0.043 | 1.23E-09 |
| rs12424086 | C | T | 0.201 | 0.999 | 0.286 | 0.054 | 1.06E-07 |
| rs4491709 | T | C | 0.715 | 0.999 | 0.126 | 0.048 | 8.10E-03 |
| rs2281845 | T | C | 0.397 | 0.956 | 0.049 | 0.045 | 0.276 |

Effect allele indicates the allele associated with lower number of primary teeth at age 15 months and longer times to first tooth. R-squared is the quality score for the imputed genotypes in MACH. Alleles refer to the forward strand. Further details on the ALSPAC GWAS for primary dentition are given in Methods S1.
