# Supplementary material for: Genome-Wide Association Study Identifies Four Loci Associated with Eruption of Permanent Teeth
Source: PLoS Genet. 2011 Sep 8;7(9):e1002275. doi: 10.1371/journal.pgen.1002275 (PMC3169538; doi:10.1371/journal.pgen.1002275)
Supplement: Table S4 — Results from GWAS of permanent tooth eruption between age 6 and 14 years in 5,104 women from the DNBC for 19 variants previously reported with P<5×10^−8^ for breast cancer in Caucasians [19], [26]–[32]. (DOC) [file pgen.1002275.s006.doc]

**Table S4**: Results from GWAS of permanent tooth eruption between age 6 and 14 years in 5,104 women from the DNBC for 19 variants previously reported with *P*<5×10^-8^ for breast cancer in Caucasians [19,26–32].

| **SNP** | **Chromosome** | **Bp** | **Effect allele** | **Other allele** | **Effect allele freq** | **Effect (SDS)** | **SE** | ***P*-value** |
| --- | --- | --- | --- | --- | --- | --- | --- | --- |
| **rs13387042*** | **2** | **217614077** | **A** | **G** | **0.510** | **-0.054** | **0.016** | **5.49E-04** |
| **rs11249433*** | **1** | **120982136** | **A** | **G** | **0.618** | **-0.042** | **0.016** | **7.74E-03** |
| **rs1562430** | **8** | **128457034** | **T** | **C** | **0.563** | **0.039** | **0.016** | **0.013** |
| rs13281615 | 8 | 128424800 | A | G | 0.628 | -0.028 | 0.016 | 0.087 |
| rs3112612 | 16 | 51192665 | A | G | 0.379 | 0.027 | 0.016 | 0.101 |
| rs2981579 | 10 | 123327325 | A | G | 0.410 | -0.023 | 0.016 | 0.143 |
| rs8170 | 19 | 17250704 | A | G | 0.206 | -0.025 | 0.019 | 0.194 |
| rs10995190 | 10 | 63948688 | A | G | 0.145 | -0.027 | 0.022 | 0.215 |
| rs889312 | 5 | 56067641 | A | C | 0.727 | 0.021 | 0.018 | 0.245 |
| rs2981582 | 10 | 123342307 | A | G | 0.388 | -0.019 | 0.017 | 0.258 |
| rs1219648 | 10 | 123336180 | A | G | 0.606 | 0.016 | 0.016 | 0.308 |
| rs865686 | 9 | 109928299 | T | G | 0.603 | -0.016 | 0.016 | 0.320 |
| rs3803662 | 16 | 51143842 | A | G | 0.250 | 0.012 | 0.018 | 0.506 |
| rs4415084 | 5 | 44698272 | T | C | 0.406 | 0.010 | 0.017 | 0.560 |
| rs3817198 | 11 | 1865582 | T | C | 0.702 | -0.010 | 0.017 | 0.579 |
| rs4973768 | 3 | 27391017 | T | C | 0.436 | 0.007 | 0.016 | 0.651 |
| rs1011970 | 9 | 22052134 | T | G | 0.168 | -0.004 | 0.021 | 0.848 |
| rs704010 | 10 | 80511154 | T | C | 0.394 | 0.000 | 0.016 | 0.978 |
| rs614367 | 11 | 69037945 | T | C | 0.145 | 0.000 | 0.023 | 0.997 |

Bold SNPs are nominally significant. An additional * indicates whether the allele associated with lower number of permanent teeth also confers increased breast cancer risk. This hypothesis is completely driven by the initial observation for rs13387042 and only holds true for one of the two other nominally significant SNPs. Alleles refer to the forward strand.
