# Supplementary material for: Genome-Wide Association Study Identifies Four Loci Associated with Eruption of Permanent Teeth
Source: PLoS Genet. 2011 Sep 8;7(9):e1002275. doi: 10.1371/journal.pgen.1002275 (PMC3169538; doi:10.1371/journal.pgen.1002275)
Supplement: Table S5 — Results for GWAS of permanent tooth eruption between age 6 and 14 years in 5,104 women from the DNBC for all 42 variants previously reported for age at menarche [33]. (DOC) [file pgen.1002275.s007.doc]

**Table S5**: Results for GWAS of permanent tooth eruption between age 6 and 14 years in 5,104 women from the DNBC for all 42 variants previously reported for age at menarche [33].

| **SNP** | **Chromosome** | **Bp** | **Effect allele** | **Other allele** | **Effect freq** | **Effect (SDS)** | **SE** | ***P*-value** |
| --- | --- | --- | --- | --- | --- | --- | --- | --- |
| **rs7821178*** | **8** | **78256392** | **A** | **C** | **0.340** | **0.064** | **0.017** | **9.63E-05** |
| **rs757647*** | **5** | **137735214** | **A** | **G** | **0.214** | **0.056** | **0.019** | **2.98E-03** |
| **rs10980926*** | **9** | **113333455** | **A** | **G** | **0.351** | **-0.040** | **0.016** | **0.015** |
| rs1361108 | 6 | 126809293 | T | C | 0.466 | 0.030 | 0.016 | 0.053 |
| rs466639 | 1 | 163661506 | T | C | 0.152 | 0.037 | 0.022 | 0.096 |
| rs7642134 | 3 | 86999572 | A | G | 0.381 | 0.027 | 0.017 | 0.099 |
| rs3914188 | 3 | 185492742 | G | C | 0.265 | 0.028 | 0.018 | 0.125 |
| rs13187289 | 5 | 133877076 | G | C | 0.185 | -0.029 | 0.020 | 0.142 |
| rs7359257 | 15 | 65489961 | A | C | 0.476 | 0.023 | 0.016 | 0.143 |
| rs10423674 | 19 | 18678903 | A | C | 0.343 | -0.023 | 0.016 | 0.150 |
| rs9939609 | 16 | 52378028 | A | T | 0.419 | 0.022 | 0.016 | 0.164 |
| rs16938437 | 11 | 46009151 | T | C | 0.099 | -0.034 | 0.026 | 0.205 |
| rs7759938 | 6 | 105485647 | C | T | 0.334 | 0.021 | 0.017 | 0.218 |
| rs12617311 | 2 | 199340810 | A | G | 0.322 | 0.021 | 0.017 | 0.225 |
| rs12472911 | 2 | 141944979 | C | T | 0.205 | 0.023 | 0.019 | 0.241 |
| rs2947411 | 2 | 604168 | A | G | 0.161 | -0.020 | 0.021 | 0.341 |
| rs6575793 | 14 | 100101970 | C | T | 0.426 | 0.016 | 0.017 | 0.342 |
| rs2687729 | 3 | 129377916 | G | A | 0.264 | -0.016 | 0.018 | 0.368 |
| rs1398217 | 18 | 43006236 | G | C | 0.419 | 0.014 | 0.016 | 0.374 |
| rs7617480 | 3 | 49185736 | A | C | 0.238 | -0.016 | 0.018 | 0.401 |
| rs6589964 | 11 | 122375893 | A | C | 0.470 | -0.014 | 0.017 | 0.415 |
| rs1862471 | 19 | 9861322 | G | C | 0.455 | -0.012 | 0.016 | 0.476 |
| rs2243803 | 18 | 41210670 | A | T | 0.426 | -0.011 | 0.016 | 0.478 |
| rs1659127 | 16 | 14295806 | A | G | 0.304 | 0.013 | 0.018 | 0.481 |
| rs6438424 | 3 | 119057512 | A | C | 0.481 | -0.011 | 0.016 | 0.488 |
| rs1079866 | 7 | 41436618 | G | C | 0.142 | 0.014 | 0.022 | 0.538 |
| rs4840086 | 6 | 100315159 | G | A | 0.437 | -0.009 | 0.016 | 0.557 |
| rs1364063 | 16 | 68146073 | C | T | 0.441 | -0.009 | 0.016 | 0.585 |
| rs633715 | 1 | 176119203 | C | T | 0.226 | 0.010 | 0.019 | 0.605 |
| rs6439371 | 3 | 134093442 | G | A | 0.335 | 0.007 | 0.017 | 0.665 |
| rs4929923 | 11 | 8595776 | T | C | 0.346 | 0.007 | 0.017 | 0.703 |
| rs10899489 | 11 | 77773021 | A | C | 0.145 | -0.009 | 0.023 | 0.713 |
| rs17188434 | 2 | 156805022 | C | T | 0.067 | -0.010 | 0.032 | 0.742 |
| rs900145 | 11 | 13250481 | C | T | 0.299 | -0.006 | 0.017 | 0.744 |
| rs9635759 | 17 | 46968784 | A | G | 0.322 | -0.006 | 0.017 | 0.745 |
| rs2002675 | 3 | 187112262 | G | A | 0.407 | 0.005 | 0.016 | 0.750 |
| rs6762477 | 3 | 50068213 | G | A | 0.440 | -0.004 | 0.016 | 0.797 |
| rs17268785 | 2 | 56445587 | G | A | 0.175 | -0.004 | 0.021 | 0.845 |
| rs3743266 | 15 | 58568805 | C | T | 0.298 | -0.002 | 0.017 | 0.911 |
| rs9555810 | 13 | 110979438 | G | C | 0.281 | 0.002 | 0.018 | 0.916 |
| rs2090409 | 9 | 108006909 | A | C | 0.333 | 0.001 | 0.017 | 0.972 |
| rs852069 | 20 | 17070593 | A | G | 0.354 | -0.001 | 0.016 | 0.977 |

Bold SNPs are nominally significant and an additional * for these SNPs indicates that the allele associated with lower number of permanent teeth has a (consistent) positive effect on age at menarche. This hypothesis is driven by the fact that permanent tooth eruption and age at menarche are weakly correlated and actually holds true for all SNPs with *P*<0.1. Alleles refer to the forward strand.
