# Supplementary material for: Genome-Wide Association Study Identifies Four Loci Associated with Eruption of Permanent Teeth
Source: PLoS Genet. 2011 Sep 8;7(9):e1002275. doi: 10.1371/journal.pgen.1002275 (PMC3169538; doi:10.1371/journal.pgen.1002275)
Supplement: Table S6 — Results from GWAS of permanent tooth eruption between age 6 and 14 years in 5,104 women from the DNBC for 180 variants previously reported for adult height [34]. (DOC) [file pgen.1002275.s008.doc]

**Table S6**: Results from GWAS of permanent tooth eruption between age 6 and 14 years in 5,104 women from the DNBC for 180 variants previously reported for adult height [34].

| **SNP** | **Chromosome** | **Bp** | **Effect allele** | **Other allele** | **Effect allele freq** | **Effect (SDS)** | **SE** | ***P*-value** |
| --- | --- | --- | --- | --- | --- | --- | --- | --- |
| **rs6473015*** | **8** | **78341040** | **A** | **C** | **0.707** | **-0.078** | **0.017** | **6.07E-06** |
| **rs1351394*** | **12** | **64638093** | **T** | **C** | **0.529** | **0.069** | **0.016** | **8.19E-06** |
| **rs1570106** | **14** | **67882868** | **T** | **C** | **0.190** | **0.065** | **0.020** | **9.17E-04** |
| **rs2597513*** | **3** | **13530836** | **T** | **C** | **0.895** | **-0.082** | **0.025** | **1.14E-03** |
| **rs425277** | **1** | **2059032** | **T** | **C** | **0.292** | **-0.047** | **0.017** | **6.17E-03** |
| **rs4640244** | **17** | **21224816** | **A** | **G** | **0.604** | **-0.040** | **0.016** | **0.015** |
| **rs9969804*** | **9** | **94468941** | **A** | **C** | **0.445** | **0.038** | **0.016** | **0.015** |
| **rs17806888*** | **3** | **67499012** | **T** | **C** | **0.892** | **0.061** | **0.025** | **0.015** |
| **rs891088** | **19** | **7135762** | **A** | **G** | **0.748** | **0.042** | **0.018** | **0.018** |
| **rs1043515*** | **17** | **34175722** | **A** | **G** | **0.465** | **-0.037** | **0.016** | **0.024** |
| **rs7763064** | **6** | **142838982** | **A** | **G** | **0.279** | **0.037** | **0.017** | **0.035** |
| **rs1013209*** | **8** | **24172249** | **T** | **C** | **0.254** | **-0.037** | **0.018** | **0.042** |
| **rs16942341** | **15** | **87189909** | **T** | **C** | **0.019** | **0.122** | **0.060** | **0.044** |
| rs720390 | 3 | 187031377 | A | G | 0.390 | 0.032 | 0.017 | 0.057 |
| rs4470914 | 7 | 19583047 | T | C | 0.177 | 0.039 | 0.021 | 0.059 |
| rs2629046 | 2 | 224755988 | T | C | 0.559 | -0.029 | 0.016 | 0.060 |
| rs2665838 | 17 | 59320197 | C | G | 0.695 | -0.031 | 0.017 | 0.070 |
| rs4605213 | 17 | 46599746 | C | G | 0.341 | 0.030 | 0.017 | 0.070 |
| rs2341459 | 2 | 44621706 | T | C | 0.263 | 0.032 | 0.018 | 0.071 |
| rs9835332 | 3 | 56642722 | C | G | 0.441 | -0.028 | 0.016 | 0.076 |
| rs7155279 | 14 | 91555634 | T | G | 0.392 | -0.029 | 0.017 | 0.081 |
| rs16964211 | 15 | 49317787 | A | G | 0.049 | -0.064 | 0.037 | 0.085 |
| rs42235 | 7 | 92086012 | T | C | 0.319 | 0.029 | 0.017 | 0.089 |
| rs4986172 | 17 | 40571807 | T | C | 0.334 | 0.029 | 0.017 | 0.090 |
| rs237743 | 20 | 47336426 | A | G | 0.233 | 0.032 | 0.019 | 0.091 |
| rs1325598 | 1 | 175058872 | A | G | 0.440 | -0.026 | 0.016 | 0.097 |
| rs1814175 | 11 | 49515748 | T | C | 0.362 | 0.027 | 0.017 | 0.102 |
| rs955748 | 4 | 184452669 | A | G | 0.243 | -0.029 | 0.018 | 0.105 |
| rs2110001 | 7 | 150147955 | C | G | 0.690 | -0.029 | 0.018 | 0.109 |
| rs12680655 | 8 | 135706519 | C | G | 0.638 | -0.026 | 0.016 | 0.112 |
| rs543650 | 6 | 152152636 | T | G | 0.402 | 0.025 | 0.016 | 0.112 |
| rs11830103 | 12 | 122389499 | A | G | 0.800 | -0.030 | 0.020 | 0.126 |
| rs13177718 | 5 | 108141243 | T | C | 0.074 | -0.044 | 0.029 | 0.132 |
| rs143384 | 20 | 33489170 | A | G | 0.567 | -0.026 | 0.017 | 0.135 |
| rs634552 | 11 | 74959700 | T | G | 0.140 | -0.034 | 0.023 | 0.140 |
| rs12982744 | 19 | 2128193 | C | G | 0.620 | -0.024 | 0.016 | 0.140 |
| rs4282339 | 5 | 168188818 | A | G | 0.215 | 0.028 | 0.019 | 0.146 |
| rs12534093 | 7 | 23469499 | A | T | 0.219 | -0.028 | 0.019 | 0.152 |
| rs1490384 | 6 | 126892853 | T | C | 0.510 | 0.022 | 0.016 | 0.160 |
| rs11648796 | 16 | 732191 | A | G | 0.756 | -0.031 | 0.023 | 0.169 |
| rs1950500 | 14 | 23900690 | T | C | 0.304 | 0.023 | 0.017 | 0.173 |
| rs17081935 | 4 | 57518233 | T | C | 0.192 | -0.027 | 0.020 | 0.176 |
| rs2871865 | 15 | 97012419 | C | G | 0.899 | -0.038 | 0.028 | 0.178 |
| rs3782089 | 11 | 65093395 | T | C | 0.072 | 0.042 | 0.031 | 0.179 |
| rs822552 | 7 | 148281567 | C | G | 0.719 | -0.025 | 0.019 | 0.187 |
| rs1351164 | 2 | 217980143 | T | C | 0.807 | -0.025 | 0.020 | 0.197 |
| rs9844666 | 3 | 137456906 | A | G | 0.252 | 0.023 | 0.018 | 0.209 |
| rs7178424 | 15 | 60167551 | T | C | 0.458 | -0.019 | 0.016 | 0.217 |
| rs7759938 | 6 | 105485647 | T | C | 0.666 | -0.021 | 0.017 | 0.218 |
| rs7567851 | 2 | 178392966 | C | G | 0.085 | 0.034 | 0.028 | 0.219 |
| rs2856321 | 12 | 11747040 | A | G | 0.650 | -0.020 | 0.016 | 0.227 |
| rs11599750 | 10 | 101795432 | T | C | 0.347 | -0.020 | 0.017 | 0.227 |
| rs1046934 | 1 | 182290152 | A | C | 0.637 | 0.020 | 0.017 | 0.229 |
| rs2778031 | 9 | 90025546 | T | C | 0.253 | 0.022 | 0.018 | 0.230 |
| rs7689420 | 4 | 145787802 | T | C | 0.153 | 0.026 | 0.022 | 0.233 |
| rs7112925 | 11 | 66582736 | T | C | 0.342 | -0.020 | 0.017 | 0.234 |
| rs572169 | 3 | 173648421 | T | C | 0.334 | -0.019 | 0.017 | 0.255 |
| rs12153391 | 5 | 171136043 | A | C | 0.264 | -0.021 | 0.018 | 0.256 |
| rs2154319 | 1 | 41518357 | T | C | 0.754 | -0.022 | 0.019 | 0.264 |
| rs5017948 | 11 | 51270794 | A | T | 0.198 | 0.021 | 0.020 | 0.280 |
| rs11205277 | 1 | 148159496 | A | G | 0.556 | -0.017 | 0.016 | 0.289 |
| rs3812163 | 6 | 7670759 | A | T | 0.541 | 0.017 | 0.016 | 0.289 |
| rs12902421 | 15 | 69948457 | T | C | 0.966 | -0.046 | 0.043 | 0.289 |
| rs1741344 | 20 | 4049800 | T | C | 0.628 | -0.017 | 0.016 | 0.293 |
| rs4072910 | 19 | 8550031 | C | G | 0.405 | -0.019 | 0.018 | 0.298 |
| rs1173727 | 5 | 32866278 | T | C | 0.431 | -0.016 | 0.016 | 0.307 |
| rs2780226 | 6 | 34307070 | T | C | 0.913 | -0.029 | 0.029 | 0.319 |
| rs3110496 | 17 | 24941897 | A | G | 0.305 | -0.017 | 0.017 | 0.322 |
| rs7507204 | 19 | 3379834 | C | G | 0.260 | -0.018 | 0.018 | 0.332 |
| rs26868 | 16 | 2189377 | A | T | 0.472 | -0.015 | 0.016 | 0.346 |
| rs7926971 | 11 | 12654616 | A | G | 0.542 | 0.015 | 0.016 | 0.347 |
| rs12694997 | 2 | 241911659 | A | G | 0.215 | 0.018 | 0.019 | 0.369 |
| rs6699417 | 1 | 88896031 | T | C | 0.593 | 0.015 | 0.016 | 0.373 |
| rs10748128 | 12 | 68113925 | T | G | 0.373 | 0.014 | 0.016 | 0.375 |
| rs2638953 | 12 | 28425682 | C | G | 0.681 | 0.014 | 0.017 | 0.389 |
| rs7864648 | 9 | 16358732 | T | G | 0.342 | -0.014 | 0.017 | 0.390 |
| rs10770705 | 12 | 20748734 | A | C | 0.335 | -0.014 | 0.017 | 0.395 |
| rs7027110 | 9 | 108638867 | A | G | 0.252 | -0.015 | 0.018 | 0.396 |
| rs751543 | 9 | 118162163 | T | C | 0.704 | 0.016 | 0.019 | 0.397 |
| rs11259936 | 15 | 82371586 | A | C | 0.496 | 0.013 | 0.016 | 0.397 |
| rs11118346 | 1 | 217810342 | T | C | 0.436 | -0.013 | 0.016 | 0.421 |
| rs806794 | 6 | 26308656 | A | G | 0.743 | 0.014 | 0.018 | 0.424 |
| rs2079795 | 17 | 56851431 | T | C | 0.323 | 0.013 | 0.017 | 0.427 |
| rs17391694 | 1 | 78396214 | T | C | 0.145 | 0.018 | 0.022 | 0.427 |
| rs2145998 | 10 | 80791702 | A | T | 0.478 | -0.012 | 0.016 | 0.430 |
| rs2336725 | 3 | 53093779 | T | C | 0.554 | 0.012 | 0.016 | 0.430 |
| rs10874746 | 1 | 93096559 | T | C | 0.377 | 0.012 | 0.016 | 0.446 |
| rs4821083 | 22 | 31386341 | T | C | 0.845 | -0.016 | 0.022 | 0.449 |
| rs310405 | 6 | 81857081 | A | G | 0.535 | 0.011 | 0.016 | 0.468 |
| rs2580816 | 2 | 232506210 | T | C | 0.175 | 0.015 | 0.021 | 0.470 |
| rs7532866 | 1 | 26614131 | A | G | 0.663 | 0.012 | 0.017 | 0.471 |
| rs2145272 | 20 | 6574218 | A | G | 0.658 | -0.012 | 0.017 | 0.474 |
| rs862034 | 14 | 74060499 | A | G | 0.369 | -0.012 | 0.016 | 0.479 |
| rs1659127 | 16 | 14295806 | A | G | 0.304 | 0.013 | 0.018 | 0.481 |
| rs12474201 | 2 | 46774789 | A | G | 0.364 | -0.011 | 0.017 | 0.490 |
| rs17318596 | 19 | 46628935 | A | G | 0.369 | -0.011 | 0.017 | 0.491 |
| rs654723 | 11 | 128091365 | A | C | 0.640 | -0.011 | 0.017 | 0.493 |
| rs8181166 | 9 | 88306448 | C | G | 0.509 | 0.010 | 0.016 | 0.509 |
| rs1046943 | 6 | 109890634 | A | G | 0.581 | 0.010 | 0.016 | 0.511 |
| rs10863936 | 1 | 210304421 | A | G | 0.507 | -0.010 | 0.016 | 0.524 |
| rs798489 | 7 | 2768329 | T | C | 0.265 | 0.011 | 0.018 | 0.531 |
| rs5742915 | 15 | 72123686 | T | C | 0.532 | 0.010 | 0.016 | 0.543 |
| rs10799445 | 1 | 225978506 | A | C | 0.805 | 0.012 | 0.020 | 0.545 |
| rs9863706 | 3 | 72520103 | T | C | 0.231 | -0.011 | 0.018 | 0.556 |
| rs4601530 | 1 | 24916698 | T | C | 0.238 | -0.011 | 0.018 | 0.557 |
| rs6470764 | 8 | 130794847 | T | C | 0.186 | 0.012 | 0.020 | 0.565 |
| rs6569648 | 6 | 130390812 | T | C | 0.777 | -0.011 | 0.019 | 0.571 |
| rs7466269 | 9 | 132453905 | A | G | 0.665 | 0.009 | 0.017 | 0.583 |
| rs7332115 | 13 | 32045548 | T | G | 0.628 | -0.009 | 0.016 | 0.591 |
| rs274546 | 5 | 131727766 | A | G | 0.393 | -0.009 | 0.016 | 0.591 |
| rs7274811 | 20 | 31796842 | T | G | 0.219 | -0.010 | 0.019 | 0.592 |
| rs9360921 | 6 | 76322362 | T | G | 0.889 | 0.014 | 0.025 | 0.593 |
| rs2093210 | 14 | 60027032 | T | C | 0.570 | -0.009 | 0.016 | 0.596 |
| rs9967417 | 18 | 45213498 | C | G | 0.579 | 0.008 | 0.016 | 0.598 |
| rs8052560 | 16 | 87304743 | A | C | 0.787 | 0.011 | 0.021 | 0.601 |
| rs9472414 | 6 | 45054484 | A | T | 0.194 | 0.010 | 0.020 | 0.607 |
| rs7909670 | 10 | 12958770 | T | C | 0.434 | 0.008 | 0.016 | 0.612 |
| rs6684205 | 1 | 216676325 | A | G | 0.721 | 0.009 | 0.017 | 0.615 |
| rs13088462 | 3 | 51046753 | T | C | 0.939 | -0.017 | 0.034 | 0.618 |
| rs6457620 | 6 | 32771977 | C | G | 0.479 | 0.008 | 0.016 | 0.629 |
| rs3118905 | 13 | 50003335 | A | G | 0.269 | 0.008 | 0.017 | 0.632 |
| rs4800452 | 18 | 18981609 | T | C | 0.796 | -0.009 | 0.019 | 0.644 |
| rs17782313 | 18 | 56002077 | T | C | 0.731 | -0.008 | 0.018 | 0.651 |
| rs7319045 | 13 | 90822575 | A | G | 0.394 | -0.007 | 0.017 | 0.652 |
| rs9428104 | 1 | 118657110 | A | G | 0.230 | -0.008 | 0.019 | 0.658 |
| rs6959212 | 7 | 38094851 | T | C | 0.302 | 0.007 | 0.017 | 0.661 |
| rs11107116 | 12 | 92502635 | T | G | 0.217 | 0.008 | 0.019 | 0.672 |
| rs2256183 | 6 | 31488508 | A | G | 0.505 | 0.007 | 0.016 | 0.676 |
| rs1330 | 11 | 17272605 | T | C | 0.341 | -0.007 | 0.017 | 0.679 |
| rs788867 | 4 | 82369030 | T | G | 0.697 | -0.007 | 0.017 | 0.687 |
| rs7853377 | 9 | 85742025 | A | G | 0.770 | 0.008 | 0.019 | 0.689 |
| rs11684404 | 2 | 88705737 | T | C | 0.620 | -0.006 | 0.016 | 0.692 |
| rs6449353 | 4 | 17642586 | T | C | 0.856 | -0.010 | 0.026 | 0.704 |
| rs1738475 | 1 | 23409478 | C | G | 0.625 | -0.006 | 0.016 | 0.708 |
| rs2279008 | 19 | 17144303 | T | C | 0.746 | 0.007 | 0.018 | 0.708 |
| rs10037512 | 5 | 88390431 | T | C | 0.558 | 0.006 | 0.016 | 0.710 |
| rs1708299 | 7 | 28156471 | A | G | 0.308 | 0.006 | 0.017 | 0.720 |
| rs1047014 | 6 | 19949472 | T | C | 0.751 | -0.006 | 0.018 | 0.721 |
| rs422421 | 5 | 176449932 | T | C | 0.203 | 0.007 | 0.020 | 0.729 |
| rs6714546 | 2 | 33214929 | A | G | 0.279 | 0.006 | 0.017 | 0.730 |
| rs1468758 | 9 | 112846903 | T | C | 0.234 | 0.006 | 0.019 | 0.732 |
| rs7567288 | 2 | 134151294 | T | C | 0.784 | 0.007 | 0.020 | 0.736 |
| rs2284746 | 1 | 17179262 | C | G | 0.481 | -0.005 | 0.016 | 0.752 |
| rs2066807 | 12 | 55026949 | C | G | 0.933 | 0.010 | 0.032 | 0.760 |
| rs11958779 | 5 | 55037656 | A | G | 0.702 | -0.005 | 0.017 | 0.763 |
| rs9456307 | 6 | 158849430 | A | T | 0.059 | -0.010 | 0.035 | 0.767 |
| rs17346452 | 1 | 170319910 | T | C | 0.711 | 0.005 | 0.017 | 0.783 |
| rs1582931 | 5 | 122685098 | A | G | 0.471 | 0.005 | 0.017 | 0.787 |
| rs3791675 | 2 | 55964813 | T | C | 0.244 | -0.005 | 0.018 | 0.802 |
| rs7697556 | 4 | 73734177 | T | C | 0.462 | 0.004 | 0.016 | 0.809 |
| rs227724 | 17 | 52133816 | A | T | 0.642 | 0.004 | 0.017 | 0.812 |
| rs4665736 | 2 | 25041103 | T | C | 0.517 | -0.004 | 0.016 | 0.812 |
| rs3129109 | 6 | 29192211 | T | C | 0.362 | 0.004 | 0.017 | 0.819 |
| rs7849585 | 9 | 138251691 | T | G | 0.329 | -0.004 | 0.017 | 0.821 |
| rs11144688 | 9 | 77732106 | A | G | 0.096 | 0.010 | 0.042 | 0.823 |
| rs2247341 | 4 | 1671115 | A | G | 0.347 | 0.004 | 0.017 | 0.826 |
| rs17780086 | 17 | 27367395 | A | G | 0.126 | -0.005 | 0.024 | 0.833 |
| rs6879260 | 5 | 179663620 | T | C | 0.383 | -0.003 | 0.016 | 0.846 |
| rs2237886 | 11 | 2767307 | T | C | 0.108 | -0.005 | 0.025 | 0.854 |
| rs1257763 | 9 | 95933766 | A | G | 0.039 | -0.008 | 0.047 | 0.859 |
| rs6457821 | 6 | 35510783 | A | C | 0.027 | 0.008 | 0.049 | 0.876 |
| rs2072153 | 17 | 44745013 | C | G | 0.326 | -0.003 | 0.017 | 0.878 |
| rs724016 | 3 | 142588260 | A | G | 0.532 | 0.002 | 0.016 | 0.886 |
| rs526896 | 5 | 134384604 | T | G | 0.716 | 0.003 | 0.018 | 0.888 |
| rs12470505 | 2 | 219616613 | T | G | 0.913 | -0.004 | 0.028 | 0.896 |
| rs473902 | 9 | 97296056 | T | G | 0.921 | 0.005 | 0.039 | 0.903 |
| rs17511102 | 2 | 37814117 | A | T | 0.919 | 0.004 | 0.032 | 0.904 |
| rs2834442 | 21 | 34612656 | A | T | 0.651 | -0.002 | 0.017 | 0.907 |
| rs6439167 | 3 | 130533446 | T | C | 0.217 | 0.002 | 0.019 | 0.914 |
| rs7971536 | 12 | 100897919 | A | T | 0.459 | 0.002 | 0.016 | 0.916 |
| rs10152591 | 15 | 67835211 | A | C | 0.903 | 0.002 | 0.027 | 0.927 |
| rs494459 | 11 | 118079885 | T | C | 0.427 | -0.001 | 0.016 | 0.932 |
| rs889014 | 5 | 172916720 | T | C | 0.341 | 0.001 | 0.017 | 0.934 |
| rs11867479 | 17 | 65601802 | T | C | 0.364 | -0.001 | 0.016 | 0.943 |
| rs961764 | 6 | 117628849 | C | G | 0.412 | -0.001 | 0.016 | 0.949 |
| rs10838801 | 11 | 48054856 | A | G | 0.682 | -0.001 | 0.017 | 0.961 |
| rs10010325 | 4 | 106325802 | A | C | 0.505 | -0.001 | 0.016 | 0.969 |
| rs3764419 | 17 | 26188149 | A | C | 0.370 | 0.000 | 0.016 | 0.979 |
| rs4965598 | 15 | 98577137 | T | C | 0.692 | 0.000 | 0.017 | 0.989 |
| rs7460090 | 8 | 57356717 | T | C | 0.896 | 0.000 | 0.025 | 0.992 |

Bold SNPs are nominally significant (*P*<0.05) and an additional * for these SNPs indicates that the allele associated with lower number of permanent teeth has a negative effect on adult height. This hypothesis is driven by the observation for chromosome 12. However, this only holds true for 6 of 12 nominally significant SNPs (leaving out rs1351394 on chromosome 12). Alleles refer to the forward strand.
