# Supplementary material for: Genome-Wide Association Study Identifies Four Loci Associated with Eruption of Permanent Teeth
Source: PLoS Genet. 2011 Sep 8;7(9):e1002275. doi: 10.1371/journal.pgen.1002275 (PMC3169538; doi:10.1371/journal.pgen.1002275)
Supplement: Table S7 — Age at menarche results for the four identified SNPs based on the GWAS meta-analysis with 87,802 women. (DOC) [file pgen.1002275.s009.doc]

**Table S7**: Age at menarche results for the four identified SNPs based on the GWAS meta-analysis with 87,802 women.

| **SNP** | **Effect allele** | **Other allele** | **Effect allele freq** | **Effect (SDS)** | **SE** | ***P*-value** |
| --- | --- | --- | --- | --- | --- | --- |
| rs4491709 | T | C | 0.706 | -0.011 | 0.008 | 0.143 |
| rs12424086 | C | T | 0.214 | -0.009 | 0.009 | 0.335 |
| rs7924176 | G | A | 0.439 | 0.004 | 0.007 | 0.548 |
| rs2281845 | T | C | 0.407 | 0.000 | 0.007 | 0.993 |

Alleles refer to the forward strand.
