# Supplementary material for: Genome-Wide Association Study Identifies Four Loci Associated with Eruption of Permanent Teeth
Source: PLoS Genet. 2011 Sep 8;7(9):e1002275. doi: 10.1371/journal.pgen.1002275 (PMC3169538; doi:10.1371/journal.pgen.1002275)
Supplement: Table S8 — Height results for the four identified SNPs based on the GWAS meta-analysis with 183,727 individuals. (DOC) [file pgen.1002275.s010.doc]

**Table S8**: Height results for the four identified SNPs based on the GWAS meta-analysis with 183,727 individuals.

| **SNP** | **Effect allele** | **Other allele** | **Effect allele freq** | **Effect** | **N** | ***P*-value** |
| --- | --- | --- | --- | --- | --- | --- |
| rs12424086 | C | T | 0.167 | - | 133,839 | 3.46E-18 |
| rs4491709 | T | C | 0.775 | - | 133,826 | 0.020 |
| rs2281845 | T | C | - | + | 133,679 | 0.581 |
| rs7924176 | G | A | 0.508 | - | 131,947 | 0.652 |

The information is limited to direction of effect, thus (-) indicates SNPs were the allele associated with fewer permanent teeth has a negative effect estimate for adult height; (+) indicates SNPs were the allele associated with fewer permanent teeth has a positive effect estimate for adult height. Effect allele frequencies are from HapMap Europeans. Alleles refer to the forward strand.
