# Supplementary material for: Genome-Wide Association Study Identifies Four Loci Associated with Eruption of Permanent Teeth
Source: PLoS Genet. 2011 Sep 8;7(9):e1002275. doi: 10.1371/journal.pgen.1002275 (PMC3169538; doi:10.1371/journal.pgen.1002275)
Supplement: Table S9 — Breast cancer results for the four identified SNPs based on the recent British Breast Cancer GWAS. (DOC) [file pgen.1002275.s011.doc]

**Table S9**: Breast cancer results for the four identified SNPs based on the recent British Breast Cancer GWAS.

| **SNP** | **N**  **cases** | **N controls** | **Effect allele** | **Other allele** | **Effect**  **allele freq** | **OR**  **Effect allele** | **95% CI** | ***P*-value** |
| --- | --- | --- | --- | --- | --- | --- | --- | --- |
| rs4491709 | 1692 | 5585 | T | C | 0.711 | 1.10 | 1.01-1.20 | 0.024 |
| rs12424086 | 1693 | 5588 | C | T | 0.205 | 1.08 | 0.99-1.19 | 0.096 |
| rs7924176 | 1692 | 5588 | G | A | 0.430 | 1.04 | 0.97-1.13 | 0.272 |
| rs2281845 | 1682 | 5586 | T | C | 0.397 | 1.04 | 0.96-1.12 | 0.379 |

Alleles refer to the forward strand.
