# Supplementary material for: Genome-Wide Association Study Identifies Four Loci Associated with Eruption of Permanent Teeth
Source: PLoS Genet. 2011 Sep 8;7(9):e1002275. doi: 10.1371/journal.pgen.1002275 (PMC3169538; doi:10.1371/journal.pgen.1002275)
Supplement: Table S10 — Kruskal-Wallis tests for data in Table 1. (DOC) [file pgen.1002275.s012.doc]

**Table S10**: Kruskal-Wallis tests for data in Table 1.

| **SNP (effect/other allele)** | **rs12424086 C/T** | | **rs4491709 T/C** | | **rs2281845 T/C** | | **rs7924176 G/A** | |
| --- | --- | --- | --- | --- | --- | --- | --- | --- |
| **Study group** | **N** | ***P*-value** | **N** | ***P*-value** | **N** | ***P*-value** | **N** | ***P*-value** |
| **Initial stage** |  |  |  |  |  |  |  |  |
| DNBC I GWAS | 5099 | 4.71E-08 | 5088 | 6.49E-09 | 5097 | 7.44E-10 | 5100 | 1.68E-07 |
| **Replication stage** |  |  |  |  |  |  |  |  |
| DNBC II | 2162 | 0.133 | 2171 | 4.43E-03 | 2172 | 1.46E-04 | 2172 | 8.07E-06 |
| DK Roskilde | 671 | 0.024 | 690 | 0.099 | 691 | 6.69E-04 | 687 | 0.185 |
| USA | 668 | 0.067 | 669 | 0.110 | 669 | 0.499 | 668 | 8.34E-03 |
| DK Glostrup | 161 | 0.221 | 161 | 0.514 | 164 | 0.025 | 163 | 0.091 |
| Replication combined | 3662 | 1.58E-03 | 3691 | 2.05E-04 | 3696 | 4.36E-03 | 3690 | 4.42E-08 |
| **All combined** | 8761 | 5.84E-10 | 8779 | 9.54E-12 | 8793 | 8.52E-10 | 8790 | 4.68E-14 |
