# Supplementary material for: Genome-Wide Association Study Identifies Four Loci Associated with Eruption of Permanent Teeth
Source: PLoS Genet. 2011 Sep 8;7(9):e1002275. doi: 10.1371/journal.pgen.1002275 (PMC3169538; doi:10.1371/journal.pgen.1002275)
Supplement: Text S1 — ALSPAC GWAS for primary dentition. (DOC) [file pgen.1002275.s013.doc]

**Methods S1: ALSPAC GWAS for primary dentition.**

*Participants and phenotypes*

Genome-wide association analyses of primary dentition data were based on data collected from 9912 children from the Avon Longitudinal Study of Parents and Children. Mothers with expected date of delivery between April 1991 and December 1992 from the Avon and Somerset region were recruited to the birth cohort. Dentition phenotypes were derived from questionnaires completed by the mothers (about the child) and include age of first tooth (assessed at 15months), number of teeth (at 15 months).

*Genotyping*

Individuals from ALSPAC were genotyped using the Illumina HumanHap550 quad chip by 23andme (The Laboratory Corporation of America, Burlington, NC, US) and the Wellcome Trust Sanger Institute (Cambridge, UK). Individuals were excluded from analysis on the basis of excessive or minimal heterozygosity, gender mismatch, individual missingness (>3%), cryptic relatedness measured by identity by decent (IBD >10%) and sample replication. Individuals were then assessed for population stratification using multi-dimensional scaling models seeded with HapMap Phase II release 22 reference populations. Individuals of non-European ancestry were removed from further analysis. SNPS with final call rate of <95%, Minor Allele Frequency <1% and evidence of departure from Hardy-Weinberg Equilibrium (*P*-value <5×10^-7^) were excluded from analysis. Of the 9912 individuals, 5,998 and 6,609 individuals had complete phenotype and genotype data for analysis of ‘age of first tooth’ (15 months) and ‘number of teeth’ (15 months) respectively. The data was imputed to HapMap Phase II (Build 36 release 22) using Markov Chain Haplotyping software (MACH v.1.0.16)

*Statistical Analysis*

Analysis of association between expected allelic dosage and ‘age of first tooth’ as measured at 15months was analysed using parametric survival analysis. The Gaussian distribution was used to model the event time. The association between expected allelic dosage and number of teeth (15 months) was analysed using proportional odds logistic regression (ordinal regression). Tooth eruption is known to erupt in pairs; hence Poisson regression (which assumes that the events of interest are independent) is not appropriate. All analyses were adjusted for age of completion (months), sex, gestational age. All data was analysed using R software package 2.9.1.
